# LIMO EEG: A Toolbox for Hierarchical LInear MOdeling of ElectroEncephaloGraphic Data

**DOI:** 10.1155/2011/831409

**Published:** 2011-02-21

**Authors:** Cyril R. Pernet, Nicolas Chauveau, Carl Gaspar, Guillaume A. Rousselet

**Affiliations:** ^1^Division of Clinical Neurosciences, SFC Brain Imaging Research Centre, SINAPSE Collaboration, University of Edinburgh, Western General Hospital, Edinburgh EH4 2XU, UK; ^2^Inserm; Imagerie Cérébrale et Handicaps Neurologiques UMR 825, 31059 Toulouse, France; ^3^Université de Toulouse, UPS, Imagerie Cérébrale et Handicaps Neurologiques UMR 825, 31059 Toulouse, France; ^4^Centre for Cognitive Neuroimaging (CCNi), Institute of Neuroscience and Psychology, University of Glasgow, Glasgow G12 8QB, UK

## Abstract

Magnetic- and electric-evoked brain responses have traditionally been analyzed by comparing the peaks or mean amplitudes of signals from selected channels and averaged across trials. More recently, tools have been developed to investigate single trial response variability (e.g., EEGLAB) and to test differences between averaged evoked responses over the entire scalp and time dimensions (e.g., SPM, Fieldtrip). LIMO EEG is a Matlab toolbox (EEGLAB compatible) to analyse evoked responses over all space and time dimensions, while accounting for single trial variability using a simple hierarchical linear modelling of the data. In addition, LIMO EEG provides robust parametric tests, therefore providing a new and complementary tool in the analysis of neural evoked responses.

## 1. Introduction

LIMO EEG (https://gforge.dcn.ed.ac.uk/gf/project/limo_eeg/) is a toolbox for the statistical analysis of physiological data. The main goal of the toolbox is the analysis and formal testing for experimental effects at all electrodes/sensors and all time points of magneto- and electro encephalography (MEEG) recordings. This contrasts with traditional approaches that select peaks or mean amplitudes of averaged evoked responses. The toolbox is implemented in Matlab (http://www.mathworks.com/) and requires the Matlab statistical toolbox (free alternative to these functions can be found on the LIMO EEG server and corresponds to adapted version of Octave functions (http://www.gnu.org/software/octave/). The data structure and visualization makes use of the EEGLAB Matlab toolbox [1] (http://sccn.ucsd.edu/eeglab/); therefore LIMO EEG is better used as a plug-in of EEGLAB, although the statistical analyses can be performed independently. Similarly, the toolbox is primarily designed for EEG data although both EEGLAB and LIMO EEG can process MEG data. 

The toolbox offers a comprehensive range of statistical tests (Table 1), including many popular designs (ANOVAs, linear regressions, ANCOVAs). Some of the statistical methods, that is, massive univariate general linear analyses [2, 3] and spatiotemporal clustering for multiple comparisons correction [4–6] have existed for several years whereas others like bootstrapping were introduced only recently [7–9]. 

**Figure 1:**
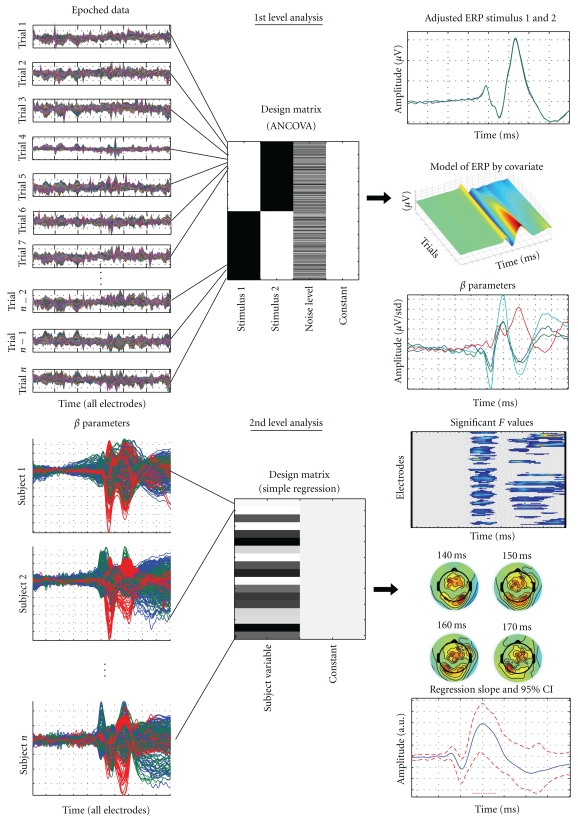
Illustration of the hierarchical procedure. At the 1st level of analysis (top), epoched data of each subject, comprising all trials, are analyzed to obtain the estimated beta parameters reflecting the effect of the various experimental conditions coded in the design matrix. Here the design is simplified from [7] and codes for the effect of stimulus 1, stimulus 2, and the noise level across all stimuli. At the 2nd level of analysis (bottom), the beta parameter(s) of experimental condition(s) coded at the 1st level are analyzed to test for significance across subjects. Here the 2nd level design matrix coded the subjects' age thus performing a regression of age on the estimated parameters that reflected the effect of noise level on visual evoked responses.

Contrary to other toolboxes dedicated to the analysis of event related potentials (ERPs), LIMO EEG deals both with within-subject variance (i.e., single trial analyses) and between-subject variance (like in e.g., SPM [2, 3]). Using LIMO EEG, data are analyzed using a hierarchical general linear model where parameters of a GLM are estimated for each subject at each time point and each electrode independently (1st level analyses). Estimated parameters from the first level analyses are then integrated across subjects (2nd level analysis—Figure 1). This hierarchical modelling of the data is similar to the one used to analyze PET/fMRI data (SPM, FSL, BrainVoyager, etc.). Our general linear approach of analyzing MEEG data thus complements others which also rely on linear modeling but focus on averaged event related data [2] rather than single trials, or factorize time [3, 8], or both, rather than using time as a natural dimension.

## 2. Method

### 2.1. Hierarchical General Linear Model for MEEG Data: 1st Level

MEEG data form 3 dimensional matrices. Following the EEGLAB convention, the 1st dimension is space (electrodes or sensors), the 2nd dimension is time and the 3rd and last dimension is trials. The analysis is performed electrode per electrode such that the data **Y** form a 2-dimensional *p*∗*n* matrix with *p* trials and *n* time frames (or time points). For each trial we define the experimental conditions by a 2 dimensional *p*∗*m* design matrix **X** with *p* rows (for trials) and *m* columns; each column codes for one condition or a covariate. In the current implementation, we consider each trial to be unique and therefore the model is similar to running a one-way ANOVA or ANCOVA. The model therefore follows (1) with **B** the estimated regression parameters (a *m*∗*n* matrix) and **E** the error term (a *p*∗*n* matrix). The solution of the normal equations is given by inverting **X**. In practice we estimate the parameters following (2), by fitting simultaneously all frames, one electrode at a time, to obtain the parameters of the univariate model on the diagonal of the **B** matrix. Combining the columns of **X** (contrast weighting) allows testing for various effects at the individual level (*t*-tests, *F*-tests—for details see, e.g., [10])

(1) $$\begin{array}{c} Y=XB+E, \end{array}$$

(2) $$\begin{array}{c} B=\mathrm{diag}\;\left({\left(X^{T}X\right)}^{-1}X^{T}Y\right)=\mathrm{diag}\;\left({\left(X\right)}^{\sim *}Y\right). \end{array}$$

In LIMO EEG, the solution is given by (2) using a generalized Moore-Penrose pseudoinverse (pinv default function in Matlab [11, 12]). Thus, although the design matrices made up by LIMO EEG are almost always rank deficient (each condition is coded in one column of *X*), *F* or *T* tests are exact, that is, they give identical results to that obtained by applying a standard inverse to a full rank matrix.

### 2.2. Hierarchical General Linear Model for MEEG Data: 2nd Level

At the second level of analysis, beta coefficients from the different conditions (or their linear combinations) obtained from each subject are analyzed across subjects to test for statistical significance. Several robust methods have been implemented in LIMO EEG at this stage. Most of the techniques described below can be found in Wilcox [13] and correspond to tests performed under H1 to compute confidence intervals and under H0 to control for multiple comparisons. Compared to standard methods, robust methods provide better probability coverage for the confidence intervals and a tighter control of the type I error. Computations presented in this section are used in LIMO EEG to provide robust confidence intervals and uncorrected *P* values or a binary decision on significance. Computations for multiple comparisons correction are presented in the next section.

#### 2.2.1. One-Sample *t*-Test

Whereas most ERP studies aim at comparing different experimental conditions, the GLM also allows testing for the covariation of single-trial ERPs with stimuli and cognitive factors (see e.g., Rousselet et al. [7] who tested the effect of image phase coherence across trials during a face discrimination task). A bootstrap-*t* approach in which subjects are drawn randomly with replacement is implemented in LIMO EEG. For each bootstrap, a one-sample *t*-test is performed on the bootstrap sample and the *T* value is stored. These *T* values provide an approximation of the *T* distribution under H0 and are used to estimate the alpha/2 and 1-alpha/2 quantiles. The *P* values are then simply the average number of times the *T* values obtained from the original data are above or below the bootstrapped quantiles. Finally, confidence intervals around the mean can be computed following (3):

(3) $$\begin{array}{cc} \text{CI} & =[\text{Sample}\;\text{Mean}-\;T_{\left(U\right)}*\frac{s}{\text{sqrt}\left(n\right)};\\ & \;\;\;\text{Sample}\;\text{Mean}-T_{\left(L\right)}*\frac{s}{\text{sqrt}\left(n\right)}], \end{array}$$

where CI is the confidence interval, *T*_(*L*)_ and *T*_(*U*)_ are the critical *T* values obtained from the sorted bootstrapped *T* values with, *L* = (alpha∗number  of  bootstraps/2) rounded to the nearest integer, *U* = (number  of  bootstraps − *L*), *s* is sample standard deviation, sqrt(*n*) is the square root of the number of observations.

#### 2.2.2. Two-Samples *t*-Test

To compare ERPs from two independent groups of subjects, we use a percentile bootstrap in which subjects from each group are sampled independently with replacement. For each bootstrap, we obtain 2 new independent samples and the mean difference between the two groups is computed. This method therefore tests differences under H1, that is, it tests that the mean of gp1 is different from the mean of gp2. After sorting these *D* differences in ascending order, confidence intervals take the values, *D*_(*L*+1)_ and *D*_(*U*)_ with *L* and *U* defined as above. If 0 is included in the CI, the difference between samples is not significant. Finally, the *P* value is the smallest value of either the averaged number of times the observed difference is above zero or, one minus this average.

#### 2.2.3. Paired *t*-Test

The comparison of two sets of estimated parameters from the same group of subjects follows the procedure described for the two-sample *t*-test. However, because data are now paired, we sample subjects with replacement, to keep pairs of ERPs together and therefore preserve the intrasubject variance.

#### 2.2.4. Regression Analysis

Regression analyses of ERP data allow assessing the inter-subject variability. Such variability is useful to test hypotheses about cognitive development, aging, various impairments, and individual differences in general (see e.g., Rousselet et al. [8, 9] for an example in normal aging). The method consists in sampling with replacement *n* matrices of ERPs (electrodes × time frames), *n* being the number of subjects. The link between subjects and predictors is maintained, so for simplicity we sample trial indices. The estimated regression parameters *β*s are computed for each bootstrap and sorted in ascending order. For a simple regression, 599 bootstraps are performed and the 95% confidence interval is [*β*_(*a*+1)_ *β*_(*c*)_], with *a* and *c* taking special values depending on the number of observations. For this simple case, 599 bootstraps have been shown to be enough to control the type I error rate [13]. For multiple regressions, a percentile bootstrap is used in conjunction with the Bonferroni inequality, and the confidence intervals are defined for each regressor as [*β*_(*a*+1)_ *β*_(*c*)_], with

(4) $$\begin{array}{c} a=\frac{\left(\text{alpha}*\text{number}\;\text{of}\;\text{bootstraps}\right)}{\left(2*\text{number}\;\text{of}\;\text{regressors}\right)},\\ c=\text{number}\;\text{of}\;\text{bootstraps-}a. \end{array}$$

No *P* value can be computed with this technique but a binary decision on the statistical significance is obtained: a regression coefficient is significant if the confidence interval does not contain 0. Compared to other techniques, the modified bootstrap has been shown to perform well under heteroscedasticity but also if the data (subjects) are sampled from a nonnormal distribution [13].

#### 2.2.5. ANOVA

Contrary to the other designs, only bootstraps under H0 are used to compute *P* values. The analysis relies on standard ordinary least squares (OLS) and, for repeated measure ANOVAs, sphericity is accounted for by a multivariate approach (Hotelling *T* ^2^ test for repeated factors and Hotelling generalized *T* ^2^ for within by between interactions; both transformed into *F* values). In a first analysis we obtain the observed *F* values. Then we build a data driven *F* table under H0. First we centre the data, independently for each group (*N*-way ANOVA) or condition (repeated measure ANOVA), so that each cell of the ANOVA has a mean of zero. Second, we use the centred data to estimate the *F* distributions under H0. We sample subjects with replacement, independently for each cell for *N*-ways ANOVAs, or keeping the association between observations in repeated measure ANOVAs. *P* values are obtained by sorting the bootstrap *F* values and counting how many times the observed *F* values are above the *F*_(*U*)_ value. Using the same resampling as above but using the original data (i.e., under H1) we also compute the average difference between conditions allowing to construct robust confidence intervals using the techniques described for *t*-tests.

#### 2.2.6. ANCOVA

The analysis of covariance follows the same strategy as the regression analysis: subjects' indices are sampled with replacement to keep data, group membership and predictors together. This resampling allows us to build robust confidence intervals around the predictors. Significance tests for the group differences and the covariate effects are obtained under H0. In this case, ERP data are sampled with replacement and fit to the original design matrix, thus breaking the relationship between the data and the predictors. We use this technique to estimate the distributions of the *F* values of group differences and covariates under H0. The *P* values are then obtained as for ANOVAs.

## 3. Multiple Comparisons Correction

**Figure 2:**
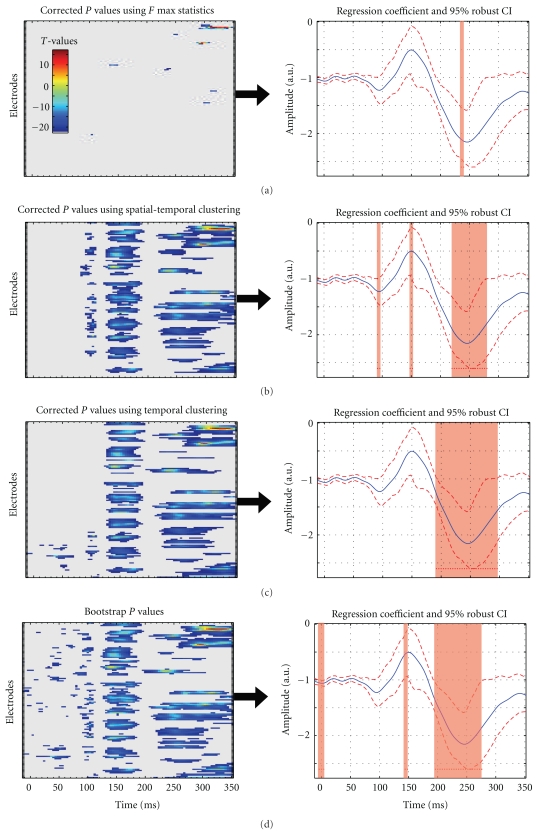
Illustration of the different multiple comparisons corrections (alpha 5%). At the top data are thresholded using a *F* max statistics (Method 1). In the middle, the same data are thresholded using spatial-temporal clustering (Method 2) or temporal clustering (Method 3). At the bottom, data are presented without any correction but a strict type I error rate (5%) for each electrode and frame separately is applied. Note that each method gives slightly different results.

Because tests are performed at many electrodes and time frames, multiple testing will give rise to a high number of false positives (type I error—see, e.g., Figure 2). This multiple comparison problem is independent of the type I error rate obtained independently at one electrode and one time frame using the techniques describe above. Computations described above were performed mainly under H1 and used for robust confidence intervals and uncorrected *P* values. These techniques are complemented here by computations performed under H0, the null hypothesis of no effect, to correct for multiple testing.

This multiple testing problem is controlled in LIMO EEG using three methods, all relying on the same bootstrap procedure. For each technique described in the previous section (*t*-tests, regression, ANOVA, ANCOVA), we sample subjects with replacement under a true (*t*-tests, ANOVAs) or estimated (regression, ANCOVA) H0. This process is repeated B times and for each bootstrap we record (1) the maximum *F* value (= *t* ^2^ for *t*-tests) among all electrodes and time frames and (2) the maximum sum of significant temporal or spatial-temporal *F* clusters. These distributions of maximum *Fs* (Method 1) and maximum *F* clusters (Methods 2 and 3) under H0 can then be used to control the type I error rate across the entire data space [14].

**Method 1 (maximum statistics)**

Uses the distribution of maximum bootstrap *F* (or *t* ^2^) values. The critical *F* value of the observed sample is corrected for multiple tests by using a probability distribution of the strongest *F* values obtained under H0 across all tests (across all electrodes and all time frames). This technique has the advantage of having an exact type I error rate [14]. However, this height threshold technique is conservative, similarly to Bonferoni and other familywise error corrections, because it is based on all the tests performed. One disadvantage of being too conservative is that, for instance, the size of a cluster of significant consecutive time frames will be smaller after correction (assuming extrema of a cluster have the lowest significant values before correction—see Figure 2) therefore possibly losing interesting information about the onsets and offsets of experimental effects. Another possibility is that this correction splits a cluster into smaller pieces because it does not take into account the spatial-temporal structure of the data. The second and third approaches use a correction based on cluster statistics and therefore overcomes this problem.

**Method 2 (spatial-temporal clustering—2D)**

Uses the distribution of bootstrap clusters defined simultaneously in space and time (Figure 2). This clustering technique follows the philosophy presented in [6] and uses functions implemented in Fieldtrip (http://fieldtrip.fcdonders.nl/). An observed spatial-temporal cluster of *F* values is statistically significant if the sum of *F* values contained in the cluster is bigger than the threshold bootstrap cluster sum obtained under H0 (see e.g., [15, 16] for a similar approach with PET and fMRI data). Under H0, one can observe by chance clusters of significant electrodes and time frames. By recording the largest sum of cluster *F* values for each bootstrap, we can construct the distribution of the spatial-temporal cluster values under H0 and therefore test the significance of an observed cluster value. Because the H0 distribution is not specific to a particular location in space and time, this technique automatically controls for multiple testing. Note that at variance with the maximum statistics, the correction only applies to clusters already declared significant, making the cluster correction less conservative. Finally, because in MEEG a large effect in, for example, size (e.g., a P300 event) can mask a smaller one (e.g., N170), the control is not performed on the cluster size itself but on the sum of the *F* (or *t* ^2^) values inside each cluster [6]. The cluster sum statistics takes into account spatial extent and height information. Therefore, a spatially narrow cluster of effects around, for example, the N170, can survive the test by a greater density of *F* values.

**Method 3 (temporal clustering—1D)**

Combines the cluster and maximum statistic approaches. For each bootstrap obtained under H0 we first take the largest temporal cluster value (sum of *t* ^2^ or *F* values) for each electrode and then only retain the largest one (Figure 2). By doing this for each bootstrap, we create an empirical distribution of temporal cluster values corrected in space. Again, an observed cluster will be significant if its sum is significantly bigger than the bootstrap threshold sum observed under H0. The advantage of this method over spatial-temporal clustering is the increased likelihood to reveal more spatially localized effects because temporal effects do not have to appear on groups of electrodes. It is also a convenient technique to test small groups of electrodes not necessarily spatially contiguous.

### 3.1. Bootstrap Computations under H0 for Multiple Comparisons Correction

The bootstrap procedures described here used the same resampling as before but often on centered data (H0 is thus true) and results are used to produce a corrected distribution (Method 1) or cluster distributions (Methods 2 and 3).

#### 3.1.1. One-Sample *t*-Test

The bootstrap procedure used to adjust the individual type I error and construct robust confidence intervals for each electrode and each frame is performed under H1. The H0 version of this bootstrap consists in centering the data and then performing one-sample *t*-tests on centred data sampled with replacement. Because centered data have a zero mean, resampling allows us to measure variations around 0, the null hypothesis.

#### 3.1.2. Two-Samples and Paired *t*-Tests

As for the one-sample *t*-test, the control of the individual type I error rates and CIs are calculated under H1 by computing differences between bootstrap group or pair samples. Therefore complementary tests under H0 are carried-out for each bootstrap. For each group or pair, data are centered and next resampled and *t*-tests computed. Because data are centered, no differences are expected (therefore testing under H0).

#### 3.1.3. Regression Analysis and ANCOVA

Subjects are randomly sampled with replacement and data are fitted to same the design matrix. This procedure thus breaks the link between the data (subjects) and the model (design matrix), and therefore allows estimating the slope(s) of the various regressors under H0. The *F* values for the different regressors or for the group effect in ANCOVAs are recorded for each bootstrap and used to compute the empirical distributions used to correct for multiple comparisons.

#### 3.1.4. ANOVAs

Here only H0 computations are performed by centering each “cell” (each group or each condition—see above). Again, recording the *F* values for each effect at each bootstrap allows correcting for multiple comparisons using one of the methods described above.

## 4. Validation

### 4.1. Low-Level Functions

In order to test the validity of the code, all statistical functions (except bootstrap and multiple comparison procedures) have been tested against Statistica and the relevant information to use each function by itself is available in a downloadable document (validation_of_the_stats.pdf) on the LIMO EEG server. For each statistical test, several low dimentional data sets have been generated and analyzed using both LIMO EEG and Statistica to ensure that LIMO EEG returns the correct *T*, *F*, and *P* values. Because of the high dimensionality of MEEG data, such analysis can not be easily carried out by standard packages and the need for multiple comparisons correction does change the statistical significance of the effects. However, this simple testing allowed us and future users to easily test the low-level statistical functions of the toolbox and be certain of our implementation. Of importance, some tests return slightly different results. The main difference can be observed for the 2 sample *t*-test. Most software (e.g., Statistica & SPSS) assume variance homogeneity by default, which is fallacious because independent groups are likely to have different variances. LIMO EEG always assumes variance inhomogeneity thus returning slightly lower *t* values. The alternative ANOVA (limo_old_rep_anova.m) which is not accessible via the interface also returns slightly different values. By default, LIMO EEG computes repeated measures ANOVA using a Hotelling *T* ^2^ test to account for sphericity. However, a standard *F* test can also be computed by changing one parameter when calling the random_robust.m function. In this case, sphericity is accounted for by a Hyund-Feld correction. The correction value is different from that of Statistica or SPSS, which use the initial formulation [17], whereas our implementation follows the modified, corrected formula [18].

### 4.2. Multiple Comparisons Correction

Permutation combined with max cluster statistics have been shown to control in theory for multiple comparisons, maintaining the probability to commit a least one type I error across the entire search space at the nominal alpha level [6]. However, permutation has not yet been validated systematically in MEEG research. Thus, despite indications that permutation performs well under certain conditions involving the comparison of two groups [6], its performance remains to be tested more generally, and its application extended to other experimental designs. Bootstrap techniques are more versatile than permutation and have been developed to address many problems in psychology [13]. For instance, it is not clear how to implement a permutation test for an ANCOVA design; whereas a bootstrap test is easy to implement. Hence, bootstrap techniques offer more possibilities to MEEG researchers. However, bootstrap techniques, and their capacity to control the type I error rate, have not yet been validated in MEEG research, which is a limitation of our toolbox. Nevertheless, we report encouraging preliminary results suggesting that bootstrap techniques perform similarly to permutation in some conditions. We compared the familywise type I error rate of permutation and two bootstrap techniques associated with max cluster statistics in *t*-tests for independent samples. 

Our simulation uses the 18 subjects of the dataset provided with LIMO and each subject was used as a “population” of about 1000 trials. This dataset is ideal to validate tests of differences under H0, because it contains ERP amplitudes spanning the whole continuum from face responses to noise responses. Thus, for each subject, we sampled with replacement from the total number of trials for that subject 100 trials twice to form fake condition 1 and fake condition 2 (level 1). Then we applied 3 tests on these 2 fake conditions. Each test involved 1000 random samples. In the first two tests, the 200 trials were pulled together and two sets of 100 trials were created either by random partitioning (permutation test), or by sampling with replacement (bootstrap test). Both tests estimate H0 by random resampling. In the third test, each group of 100 trials was mean centred and bootstrap samples with replacement drawn independently from each of them (technique implemented in LIMO and validated in [13]). For each test, and for each random sample, a *t*-test was performed to compare the groups of trials, followed by spatial-temporal clustering of *F* values (squared *t* values). We cluster the *F* values because a *t*-test is a special case of linear contrast, which is evaluated using an *F* statistics. Also, an *F* statistics is used for all the other GLM designs. Then we saved the maximum *F* cluster sum, and obtained a distribution of max cluster sums under H0, which was used to assess the significance of the original *t*-tests. So far we have conducted level 1 H0 analyses 200 times on each subject. The average number of positive tests is the type I error rate, after correction for multiple comparisons using cluster statistics. Across 18 subjects, and using 200 simulations, the type I error rate for permutation is 0.0506, with minimum 0.025 and maximum 0.085 across subjects. The type I error rate for the bootstrap test is 0.0489, min = 0.025, max = 0.08. The type I error rate for the bootstrap test with data centering is 0.0453, min = 0.025, max = 0.08. These results are very close to the nominal alpha results of 0.05. More simulations and more situations will need to be tested to compare precisely the behaviour of these techniques.

## 5. Graphical User Interface

**Figure 3:**
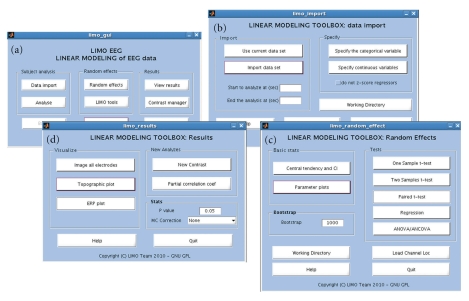
The four main GUI of LIMO EEG. All functions and plots are available via these user interfaces.

LIMO EEG can be called directly in the Matlab command window or via the EEGLAB menu. It comes as a fully functional graphical user interface (GUI). Each of the main steps have there own GUI: General GUI (Figure 3(a)), import of epoched data and 1st level analysis (Figure 3(b)), 2nd level analysis (Figure 3(c)), and visualization (Figure 3(d)). User do not have to call functions or type anything in the command window, everything can be obtained via interface. Each time a help button is also available for a description of each option in each GUI. In addition, we made available a data set on the LIMO EEG server which comes with a tutorial explaining how to analyse the data using there interfaces. A short example of results is given in the next section.

## 6. Application to EEG Data and Visualization Tools

In this section, we present some results from an analysis performed on 18 subjects to illustrate the various formats in which group data can be explored and presented. This data set is downloadable as a tutorial for LIMO EEG and results represent simplified analyses of what is presented in [8]. In short, subjects of various ages discriminated between pictures of two faces, face A and face B; the noise level in the images was varied parametrically (actually a manipulation of the phase of the image). Using such design one can therefore test for differences between ERP to the two faces using a paired *t*-test, test for an effect of the noise level using a one-sample *t*-test, or test for an age effect on ERP noise sensitivity using regression analysis. 

### 6.1. 1st Level Analysis

For each subject we create a design matrix including face A, face B and the level of image noise (see Figure 1 top). The data are thus modelled as a weighted sum of three predictors (face A, face B and phase coherence effect) plus a constant and an error term.

### 6.2. 2nd Level Analysis

**Figure 4:**
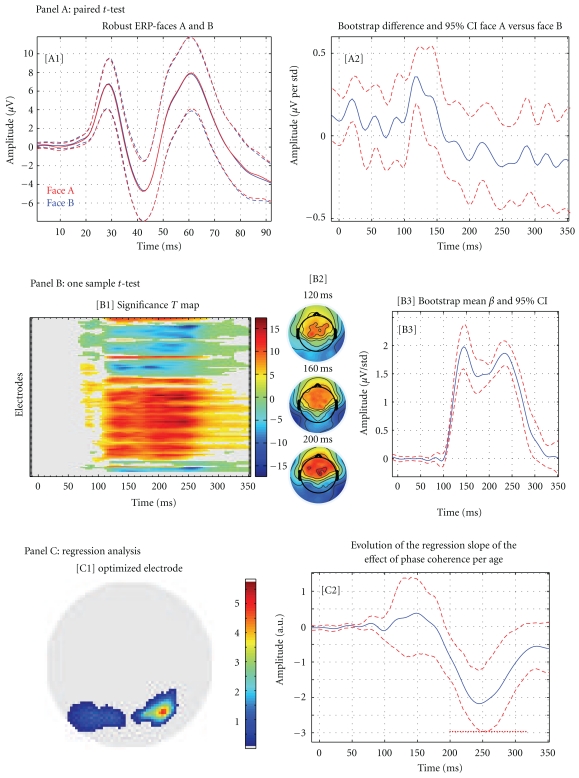
Examples of analyses/results obtain with LIMO EEG. Panel A presents results from a paired *t*-test between Face A and Face B (A1: average of the 20% trimmed means ERPs for face A and face B; A2: mean difference of estimated beta parameters and robust 95% confidence intervals). Panel B presents results from a one-sample *t*-test performed on the phase coherence regressor (B1: map of significance over all electrodes and frames; B2 topographic projection of the *T* values around the N170 event, B3: mean beta parameter (phase coherence) and robust 95% confidence interval for the electrode showing the strongest effect). Panel C presents results from the regression analysis of age on the phase coherence regressor (C1 map for the optimized electrode, that is, the map represents the location of the electrode chosen for each subject and the number of subjects; C2 results of the regression analysis, that is, plot the regression slope of the effect of phase coherence (estimated beta parameters) per subject age).

Using the estimated parameters from each subject one can test several effects. First, we looked for differences between faces A and B using a paired *t*-test (no significant effects, *P* = .05 corrected with spatial-temporal clustering) by entering into the analysis the estimated beta parameters for face A and face B from all subjects. As illustrated in Figure 4 (panel A), face stimuli evoked a typical ERP (A1, tools are provided to plot robust ERPs across subjects, here the average of 20% trimmed mean ERPs with 95% CI obtained using the bootstrap standard error) and no significant differences can be observed (A2). Second, we investigated a possible effect of the stimulus phase coherence on the visual evoked response. This was performed using a one-sample *t*-test (*P* = .05 corrected with spatial-temporal clustering) by entering the estimated beta parameters corresponding to this predictor. As illustrated (Figure 4—panel B), image phase coherence affects the evoked brain responses from 80 ms onward (full space/time map—B1) mainly over posterior lateral and central electrodes (topographic plot of *F* values—B2) with the strongest effect observed on electrode C1 between 110 ms poststimulus onset and 290 ms (B3). Finally, we also investigated an effect of age on ERP phase sensitivity by performing a simple regression with age as covariate (Figure 4 panel C, *P* = .05 corrected with spatial-temporal clustering). This analysis could be performed over the whole scalp by taking the same physical electrodes across subjects (data are presented Figure 2). The analysis can also be performed using an optimized electrode [19]. This strategy consists in selecting the electrode that shows the strongest model fit, so that we compare functionally similar electrodes across subjects. In this case, the analysis of the age effect on ERP sensitivity to noise was performed on the electrode that best modelled the data in each subject, as defined by the strongest *R* ^2^ (C1). This feature of LIMO EEG allows more flexibility in the way one combines data for group analyses. Here, results show the ERP sensitivity to image phase coherence is significantly modulated by age from 200 ms to 330 ms post stimulus onset (C2).

## 7. Discussion

### 7.1. Pros and Cons of a Massive Univariate Approach in MEEG

LIMO EEG relies on a massive univariate approach in which, like PET or fMRI, all possible measurements (voxels or electrode/time frames) are analyzed. This provides many advantages but also elicits some problems. On the positive side, the massive univariate approach is relatively easy to understand as it uses standard statistics, it is fully automatic, accommodates any design, and provides a full picture of electromagnetic events without having to hand pick electrodes or time frames. On the negative side, strict controls of statistical tests need to be implemented because of the multiple tests performed. Also, because analyses are performed on independent electrodes and time frames, one can miss more subtle effects that might develop over time or space, and would be picked up by multivariate [20] or multidimensional [21] approaches. However, the down side of these latter approaches is that they are much harder to interpret.

### 7.2. Robust and Parametric

In LIMO EEG, as in any parametric statistics package, we assume data come from a type of probability distribution, and makes inferences about the parameters of these distributions. In LIMO EEG, we assume that data come from a normal or nearly normal distribution, and make an inference about the mean values. Another important feature of LIMO EEG is the use of robust statistics. Here “robust” is used in the sense that the techniques implemented in LIMO EEG show overall more power than traditional tests when assumptions (e.g., normality) are violated and when experiment effects exist (H1) thus providing better probability coverage, especially when estimating confidence intervals. Using those techniques, we also ensure a tighter control of the type I error rate (H0). Our preliminary simulation results (18 times 200 Monte-Carlo) show that using 1000 bootstraps, the mean type I error rate of our 2 samples *t*-test is 0.0453, demonstrating that the cluster technique for multiple comparison correction offers a good control on false positives. Further simulations are needed to adequately test the type I error rate in various situations (designs/population) but this demonstrate, in principle, the validity of our method. 

In LIMO EEG we limited the scope of most analyses to samples' means via bootstrap. In fact, robust statistics allow analyzing data using various distribution estimators other than the mean. The mean is not necessarily a good estimator of the central tendency of the data, and trimmed means, median, and M-estimators can provide more satisfactory results [22, 23] (there are trimmed means options in LIMO EEG and a few stand-alone functions to do, for example, *t*-tests on trimmed means). However, none of these estimators have been validated for MEEG data yet, hence the restriction to samples' means. 

One current limitation of our parametric approach is that first level analyses, and the GLM designs at the second level, currently rely on an OLS solution. Ideally, one can make regressions more robust using weighted least squares (WLS). However, the problem of WLS is the computation of the covariance matrix. If one wants to properly estimate how trials/conditions (1st level) or subjects/conditions (2nd level) covary, new methods must be investigated in order to account for the spatial and temporal link between data points and not merely the covariation between conditions/subjects at each time point separately. Until such a method is available, an OLS solution seems the safest option.

### 7.3. Current Limits and Future Development

There is no real limit to the current implementation of LIMO EEG because it allows analyzing almost all kinds of designs. Limits are only related to various statistical aspects that deserve consideration. One current limit concerns the 1st level of analysis: all conditions are treated independently, which effectively corresponds to a 1 way ANOVA or a 1 way ANCOVA. However, experimental conditions could also be grouped in order to create a factorial design, thus pooling some variances together to account for interaction effects. Although our approach is valid because the estimated parameters of each condition can be combined via contrasts to reflect main effects and interactions as in a factorial design, it is likely to limit some analyses. Therefore, future versions of the toolbox will incorporate factorial variance pooling. A second limitation is the use of OLS. As mentioned above (*Robust and parametric section) *one would ideally use a WLS solution to allow non independence and heteroscedasticity between conditions. However current mathematical solutions do not exist to properly estimate the covariance matrix and until then the 1st level estimates will not be “robust”.

## 8. Conclusion

Overall LIMO EEG provides a set of statistical tools allowing the analysis of many designs via GUI. It provides robust results which are unbiased by the selection of peaks or components. It also provides a new way to analyze data with an emphasis on effect size (robust confidence intervals), which we hope will help moving the field toward a more quantitative analysis of evoked neural responses [7].

## Figures and Tables

**Table 1:** Summary of statistical tests available in LIMO EEG via the GUI with the bootstrap procedures used at the univariate (one time frame on one electrode) and cluster levels.

Statistical tests available via the general user interface	Hypothesis tested at the univariate level using bootstrap (non corrected *P* values/significance testing)	Multiple comparisons correction (accounts for doing many tests) using cluster statistics
One sample *t*-test (Student *T* test)	H1 (resample subjects and use bootstrapped *T* values)	H0 (center data then resample subjects and use bootstrapped *T* values)
Paired *t*-test (Student *T* test)	H1 (resample subjects paired observations and use bootstrapped *T* values)	H0 (center data per condition then resample subjects and use bootstrapped *T* values)
Two samples *t*-test (Student *T* test)	H1 (resample subjects in each group and use bootstrapped mean differences between groups)	H0 (center data per group then resample subjects and use bootstrapped *T* values)
Regressions (Fisher *F* test)	H1 (resample subjects and use regression coefficients)	H0 (resample subjects and fit to the original design matrix and use bootstrapped *F* values)
One way ANOVA (Fisher *F* test)	H0 (center data per group then resample subjects and use bootstrapped *F* values)	H0 (center data per group then resample subjects and use bootstrapped *F* values)
One way ANCOVA (Fisher *F* test)	H0 (resample subjects and fit to the original design matrix and use bootstrapped *F* values)	H0 (resample subjects and fit to the original design matrix and use bootstrapped *F* values)
Repeated measures ANOVA (Hotelling *T* ^2^ test)	H0 (center data per conditions then resample subjects and use bootstrapped *F* values)	H0 (center data per conditions then resample subjects and use bootstrapped *F* values)
